# Insufficient Sleep Syndrome in Children and Adolescents

**DOI:** 10.3390/children13060803

**Published:** 2026-06-11

**Authors:** Jun Kohyama

**Affiliations:** Tokyo Bay Urayasu Ichikawa Medical Center, 3-4-32 Toudaizima, Urayasu 279-0001, Chiba, Japan; info@j-kohyama.jp

In this Special Issue of Children, entitled “Insufficient Sleep Syndrome in Children and Adolescents”, recent advances in pediatric sleep medicine are explored through several diverse and thought-provoking perspectives on insufficient sleep syndrome (ISS) across developmental stages and clinical contexts. Collectively, these contributions emphasize that ISS should not be regarded merely as a reduction in sleep duration, but rather as a complex biopsychosocial condition with broad implications for neurodevelopment, behavior, mental health, and quality of life.

Among the articles included in this issue, Hirata et al. [1] highlighted the strong association between irregular sleep schedules, ISS, and school non-attendance, thereby emphasizing the significant social and educational consequences of chronic insufficient sleep in children and adolescents. Tanaka et al. [2] further demonstrated that improving ISS requires not only knowledge acquisition, but also structured behavioral support and intervention strategies capable of facilitating actual lifestyle modification. Houshialsadat et al. [3] approached ISS from the increasingly important perspective of the gut–brain axis and suggested that sleep-related alterations in the gut microbiota may represent a novel biological dimension of insufficient sleep in children. These findings are consistent with growing evidence demonstrating that adolescent sleep insufficiency is strongly influenced by circadian delay, social schedules, academic demands, digital media exposure, and behavioral sleep patterns [4,5,6,7,8,9,10,11,12], while successful intervention requires long-term behavioral reinforcement and school-based support [13,14].

Recent evidence increasingly supports the concept that sleep and the gut microbiota interact bidirectionally through neural, endocrine, immune, and metabolic pathways. Sleep deprivation and circadian disruption have been associated with alterations in microbial diversity, intestinal permeability, inflammatory signaling, and neurobehavioral regulation through the microbiota–gut–brain axis [15,16,17]. Conversely, gut microbial dysbiosis itself may contribute to sleep fragmentation, circadian instability, emotional dysregulation, and daytime dysfunction through modulation of neuroimmune and neuroendocrine pathways [15,16,17]. These findings suggest that the gut–brain axis may provide an important mechanistic framework for understanding the biological consequences of chronic insufficient sleep and may become a future therapeutic target for pediatric sleep disturbances.

Taken together, these articles substantially deepen our understanding of ISS as an increasingly prevalent pediatric health problem. As editors of this Special Issue, we would also like to emphasize one particularly important issue regarding ISS that has received insufficient attention in previous research. Traditionally, many studies have defined insufficient sleep solely according to sleep duration. For example, sleeping less than six hours is often automatically categorized as “sleep deprivation”. However, optimal sleep duration (OSD) varies considerably among individuals. If one individual’s OSD is seven hours and another’s is nine hours, then eight hours of sleep would not constitute insufficient sleep for the former, whereas it may still represent sleep insufficiency for the latter. Although this point may appear self-evident, it has largely been overlooked in previous studies. We believe that incorporating individual variability in sleep need into future ISS research will be critically important for advancing both scientific understanding and clinical practice.

In addition, recent clinical practice increasingly tends to equate excessive daytime sleepiness (EDS) with narcolepsy. However, EDS is a nonspecific symptom, and insufficient sleep is among its most common and underestimated causes [18]. Particularly in children and adolescents, chronic sleep restriction associated with academic demands, social schedules, digital media exposure, and delayed sleep phase tendencies has become increasingly prevalent [4,5,6,7,8,9,10,11,12,19].

Importantly, insufficient sleep itself may produce findings overlapping with central hypersomnolence disorders. A substantial proportion of patients with ISS have been shown to exhibit sleep-onset REM periods (SOREMPs) on the Multiple Sleep Latency Test (MSLT), potentially complicating differentiation from narcolepsy type 1 (NT1) [20]. Furthermore, actigraphic analyses demonstrated that ISS is characterized by marked weekday sleep restriction with weekend catch-up sleep, whereas NT1 more typically shows fragmented nocturnal sleep and frequent daytime naps [20]. These findings indicate that EDS alone should never be regarded as synonymous with narcolepsy and underscore the importance of carefully evaluating habitual sleep duration, weekday–weekend variability, and sleep–wake behaviors before establishing a diagnosis of central hypersomnolence disorders.

Overall, this Special Issue highlights the need to move beyond symptom-based diagnostic frameworks toward a broader understanding of sleep health literacy, behavioral sleep patterns, socially driven sleep insufficiency, and chronobiological vulnerability in contemporary youth. Future studies should integrate chronobiology, behavioral science, neurodevelopment, school-based intervention research, and gut–brain axis science to clarify the biological and psychosocial mechanisms linking chronic insufficient sleep to emotional dysregulation, cognitive dysfunction, and hypersomnolence, while also establishing more precise diagnostic frameworks and effective intervention strategies for ISS in real-world pediatric clinical settings.
